# Resisting Psychopathologies of Dominance and Authoritarianism: From Trumpian Dystopia to Better Tomorrows

**DOI:** 10.1111/jpm.70132

**Published:** 2026-04-29

**Authors:** Michael Haslam, Mick McKeown

**Affiliations:** ^1^ School of Nursing & Midwifery, University of Lancashire Preston UK

## Abstract

**Background:**

The world and mental health nursing face several crises that, in different ways, reflect problems of dominance. Global politics are afflicted with a growth of support for right‐wing ideologies associated with domineering authoritarian leaders. Mental health services are dominated by a singular application of bio‐psychiatric ideas and practices. Both forms of dominance are potentially alienating and harmful.

**Argument:**

Psychosocial theories offer ways to make sense of authoritarian tendencies across both realms and point to how dominance can be resisted towards progressive transformation. Authoritarian right‐wing politics arguably pose an existential threat to society, which is implicated in growing mental distress at the population level and damages services by squeezing resources. Dominant bio‐psychiatry visits epistemic and material harms upon the mentally distressed and restricts choice over care and support. Nurses operate at the relational and political nexus of these harms, so must be able to make sense of generative factors and be active in providing remedies.

**Conclusions:**

Applying psychosocial theory can raise political consciousness for mental health nurses and support development of a new politicised professional identity. Advocacy and activist‐oriented nurses can mitigate harms across societal and practice‐level political engagement. We can, and arguably must, be part of resistance movements grounded in a relational ethic of care.

## Accessible Summary

**Originality:**

- Theories of dominance are examined to provide insights into new forms of fascism and problems facing mental health services.
- The worlds of mental health practice and wider politics collide in the work of mental health nurses.
- We argue that nurses must be part of a resistance to dominance in all its forms.
- This should involve a rethinking of nursing professionalism.

**Significance:**

- The problem of dominance is substantial in society and mental health services. This is harmful for citizens, service users and staff.
- Mental health nurses need to better understand the impact of right‐wing politics as a cause of mental distress and as responsible for cuts to services.
- Thinking about professionalism in political terms will enable nurses to help shape better services and a better world.

**Rigour:**

- Relevant theory has been thoroughly reviewed with alertness to different perspectives and arguments
- The central argument is closely grounded in theory and is built up in a logical order.
- Care has been taken to demonstrate relevance to mental health nursing and core professional values.

## 1 Introduction

This paper offers a critical examination of aspects of contemporary politics through a psychosocial lens, with a particular focus on the growth of right‐wing authoritarianism, exemplified by Donald Trump but not exclusive to him. These political developments are considered indicative of a new wave of fascism. We draw some parallels with the psychosocial realm of mental health services, specifically for individuals who carry a diagnosis of personality disorder but arguably have broader resonance across an epistemically dominant bio‐psychiatry. We suggest that insights drawn from previous critical psychosocial studies and more recent phenomenological analyses of mental health services offer a means for sustaining resistance predicated on clarifying understanding of the nature of these problems. Such resistances can be framed as part of a progressive, politicised nursing professionalism: of potential value in the everyday democracies of relational practices with service users, team relations and the broader democracy of society. The central argument focuses on challenging the pervasive and detrimental consequences of dominance, making the case that mental health nurses have a key role to play in resistance.

Not all right‐wing thinking is fascistic or authoritarian, and the left also has not been historically averse to totalitarianism, so we must have precision in our objections. Similarly, not all psychiatry is oppressive, and some critics can be guilty of overly blunt reasoning or unfair personalized attacks. That said, under neoliberal capitalism there has arguably been accelerated oppression and disadvantage at the expense of social cohesion, and the dominance of an overly coercive set of psychiatric practices continues to be sufficiently pervasive to be objectionable.

We must also take care to admit that incipient or overt fascism is not too simplistically a function of psychopathology; there is something more complicated at stake. Historian commentators such as Lasch (see Blake and Phelps 1994) have pointed out the problem of over‐emphasizing psychiatric categories in contemplation of the political realm, if this proves a distraction from engaging in critical debate and argumentation over ideas. That is, framing analyses around psychopathology can excuse the hard work of politics if the opposition can be too readily dismissed as exhibiting forms of madness. (In any event, autocrats like Trump can seem relatively immune to this sort of criticism). As well as all the seeming irrationality there is almost certainly a cold (even cynically calculating) rationality to the rise of the nouveau right‐wing and attendant oppressions. We conclude with suggestions for resistances on all fronts that are deeply democratised and relational.

Our thesis reaches for explanatory ideas that connect with various psychosocial theorists, with reference to Frankfurt School scholars such as Marcuse and Fromm. Our intention here is not to suggest that Trump or other authoritarian leaders are mentally ill (they are seemingly not personally bothered or distressed by their demonstration of psychopathological traits) (Frances 2017). In this regard, we hope to avoid: ethical traps (Bandy 2017; Pouncey 2018), reification of the dominance of psychiatry and diversion from legitimate mad liberation struggles (White 2022), or adding to stigma (Smith‐Frigerio and Houston 2018). Instead, our purpose is to present a psychosocial understanding of the interplay between authoritarianism and its societal support, with a view to being better able to dismantle it utilising a democratic relational politics suited both to transformations of mental health care and society at large (see Dyson 2024; Sedgwick 1982). We argue a specific case for mental health nurses to become active in such resistances. We start with thinking about Donald Trump as a particular example of authoritarian leadership.

### 1.1 Trumpist Authoritarianism

There are several national exemplars of the rise of right‐wing demagogic, authoritarian leaders. Arguably, Donald Trump in the US is the archetypal example. It is difficult now to avoid the presence of Trump as he projects his personality at home and across the globe. This aggressively posturing statecraft, now warcraft, − comprising dominance and authoritarianism wrapped up in toxic masculinity—poses real threats to democracy and well‐being worthy of consideration in the pages of this journal. We contend there is scope to understand this, and hope for resolving consequential detriments, with recourse to psychosocial theories. In doing so, we acknowledge some caveats. For instance, there is a danger of over‐pathologising disliked political practices in seeming contradiction of our preferred critical standpoint towards aspects of the social role of psychiatry and psychology, including unease surrounding psychiatric terminology and labels. That said, ironically, there is some political appeal in highlighting notions of personality disorder when contemplating the behaviour of a president and autocrat who to all intents and purposes defines himself in terms of his personality and self‐conception; unashamedly celebrating these traits in a MAGA (Make America Great Again) cult of personality.

Arguably, the sort of politics associated with Donald Trump's presidency, and other demagogic leaderships elsewhere, pose existential threats to, amongst other things, mental wellbeing at a population level. Faced with the attendant rampant military interventionism abroad, including genocide in Gaza and the bombing of Iran contrary to international law, the weaponisation of policing at home including demonisation, dehumanisation and forced deportations of migrants, an oppressive and deadly response to protest and various attacks on the process and institutions of democracy, it is somewhat easy to become desensitised and demoralised, as well as fearfully anxious about where this will all end. In a context of increasing concentration of wealth in the hands of a few and ongoing immiseration of the many, particularly the minoritised, it is no surprise to see there is an epidemic of anxiety in the world (see Mark Fisher 2009). We now turn to consider aspects of Trump and his supporter's behaviour in terms of psychopathology, including acknowledging that such a turn can be problematic.

## 2 Trump and His Supporters: A Problem of Personality?

A number of contemporary commentators have attempted to understand Donald Trump through a psychological or psychosocial lens (e.g., Burston 2020). From these perspectives the Trump psyche is characterised by extreme self‐serving and domineering extroversion and grandiosity, low agreeableness and off the scale narcissism. Such traits are arguably commensurate with narcissistic personality disorder. In the extreme, it is possible to detect notions of malignant narcissism, a hypothesised state where narcissism merges with paranoia and antisocial or psychopathic tendencies (Semel 2023). More detailed character analysis notes how this congruence with diagnostic criteria aligns with Trumpian behaviour such as a propensity to take credit for success and blame others for failure, including taking credit for achievements rightly due to others, and an obsessive focus on winning. His preferred leadership style shows a lack of empathy and a proclivity for bullying.

As such, he is drawn to dominance in both personal and geo‐political relationships, within which he is remarkably impulsive and inconsiderate of standard protocols for civility and diplomacy. Additionally, he has been found guilty of serious financial and sexual criminality, inciting insurrection and is closely associated with Jeffrey Epstein's criminality. In everyday behaviour, Trump is undoubtedly a compulsive liar and rampant misogynist, with a host of unpalatable ties to racists and white supremacists. This has led to increasing consensus that he presides over a fascist politics; this despite most commentators on the left being acutely aware of the hazards of over‐zealously identifying fascism. One such signifier, amongst many, is Trump's mobilisation of negative emotions like fear and anger to boost his populist support.

If we are to consider Trump in malignant psychopathological terms, are we then to treat his supporters in the same way? This is potentially a more contentious leap, but it is worth thinking through questions regarding why an authoritarian neo‐fascism does appear to have some popular appeal. From a psychosocial perspective, the psychology of Trump's electoral and MAGA activist support needs to be understood in terms of the appeal of authoritarianism. This can be psychologically linked to desires for a distinct form of strong leadership that are in turn predicated upon the insecurities of denuded economic and cultural circumstances associated with globalisation and de‐industrialisation, the stoking of prejudice linked to in‐group/out‐group identities and associated othering. This builds up to an increasing focus on perceived threats, a need for order and loyalty to a demagogic leader who promises restoration of past glories and prosperity. Such appeal is coupled with wilfully blaming a combination of established democratic institutions and demonised scapegoats, such as people of migrant heritage, for all prevailing social ills. In this way, there is little need for a clear political analysis or suite of well thought out policies, as these can be substituted by a more simple and atavistic appeal framed around the domineering personality of the leader.

In the US, sections of a disadvantaged white working class and equally white business class offer immediate support, the former being oblivious to their exploitation by the latter because it becomes easier to blame the scapegoats in the absence of meaningful politics. This simplicity has arguably been amplified in the US because of long histories of racism and racist violence going back to slavery and Jim Crow. In such a political climate, right wing support for Trumpian authoritarianism goes psychologically hand in hand with a yearning for one's own social group to achieve dominance. In the extreme, this takes the form of white supremacy (Bussaja 2024). More prosaically, an implicit racism runs through the forms of othering necessary to sustain the belief that minorities are to blame for wider economic misfortunes. The MAGA movement arguably displays all the features of a personality cult, where the ‘strong’ leadership of Trump, and him alone, seems the only solution to perceived problems and inspires cultish devotion and forgiveness, or even appreciation, of despotic, domineering tendencies (Diamond 2025). Association with this strong‐man image helps to bolster against the personal insecurities and inadequacies of the ‘cult’ members and ameliorate a previous sense of helplessness or impotence in the face of prevailing economic and cultural changes. Various scholars, often in consideration of key moments in history and tyrannical historical figures, have contributed to understanding the rise of authoritarian regimes. It is to these critical thinkers that the essay now turns.

## 3 Marcuse and the Authoritarian Personality

The second generation of Frankfurt School scholars notably demonstrated a shift from earlier political pessimism as they attempted to navigate ideologies of progress for lifting society and human relations out from under the baleful prospect of dominance. Notable amongst this group were thinkers such as Marcuse and Fromm, who forged connections between critical theory and new‐left politics in the 1960s. Formed in a context of political crisis, arguably, these analyses of authoritarianism hold persuasive power to this day (Laubender 2026).

Herbert Marcuse's psychosocial analysis of modern industrial society posits that a psychopathology of authoritarian personality is grounded in the creation of surplus repression. This leads individuals to embrace domination. From this point of view, authoritarianism is both a political structure and entrenched in the social psychology of individuals, with irrational, libidinal attachments to authority as a means to discharge repressed resentment. In a psychosocial sense, libidinal attachment refers to an investment of psychic energy. In ordinary human relationships, for example, this explains investments of love between people. But in this context what we see is deference and subjugation to domineering authority, becoming a convenient mechanism for fueling far‐right populism and authoritarianism (Marcuse 1964).

For Marcuse (1955), other interlinked psychosocial ideas help to further explain social approval of authoritarianism. Working with notions of sublimation, the processes by which socially unacceptable impulses are transformed into more approved of behaviours, Marcuse considered ways in which the reverse can be played out within society. Repressive de‐sublimation is how arguably higher human aspirations such as art, sexuality and thought are displaced by a more shallow and immediate set of gratifications offered by a consumer society and dumbing‐down of mass culture. The inner‐worlds of people under a techno‐rational capitalism become dominated by a culture that is increasingly one‐dimensional, thus overpowering the capacity to imagine alternatives and neutralising dissent (Marcuse 1964). The status quo becomes taken for granted as an almost natural way things are.

Writing at the time of Richard Nixon's re‐election, Marcuse and Kellner (1972: 165) revisited the pessimism of the first wave of Frankfurt School scholars to argue that right‐wing, fascistic authoritarianism may be the inevitable result of failings of western democracy:

The historical fate of bourgeois democracy: its transformation from a dynamic into a static, from a liberal‐progressive into a reactionary‐conservative state. This democracy has become the most powerful obstacle to change – except change to the worse.

On this basis, Marcuse foresaw the rise of Trump, envisaging the rise of a new type of authoritarianism, marked by irrationality and a disregard for logical discourse. Marcuse was also prescient in questioning the beneficence of technologies, arguably offering a convincing analysis of contemporary relevance. This being acutely relevant to anxieties associated with new technologies and surveillances, such as how the misuse of the algorithms of artificial intelligence and social media platforms might underpin authoritarian dominance.

## 4 Fromm and Authoritarianism as Sadism

Erich Fromm made connections between sadism and authoritarianism as malignant forms of aggression; where sadism is part of an authoritarian character which is often associated with the desire for absolute control, seeking to master another living being by reducing them to mere instruments without will of their own (Fromm 1937, 1957). Through this lens, MAGA can be understood in terms of psychosocial mechanisms rooted in human destructiveness and desires to minimise anxiety associated with feelings of alienation, desperation, powerlessness and isolation. Any sense of strength or freedom, however, is illusory: sadists appear to be confident, but this actually conceals a deep sense of impotence. They try to feel strong by ‘devouring’ or controlling others.

Importantly, Fromm strongly contrasts sadism with genuine love, with the latter being based on equality, freedom and respecting the integrity of others. Conversely, authoritarian rule as a form of sadism is a perversion of power, masquerading as love or care, but actually representing a negation. Later sociologists, such as Brian Easlea, have emphasised how Fromm's analysis intersects with particular notions of masculinity, and these observations are also evident in contemplation of Trump. In this regard, Fromm notes how a rise of authoritarianism and sadism, for instance in the fascisms of the 20th century, are connected to ideas of patriarchal dominance: where control over others (specifically women and minorities) became a key component of the male social character. Interestingly, Trump and his government have also advocated fascist‐like policies, akin to the eugenics inspired Nazis, in relation to health care, alongside specific sweeping funding cuts for mental health and substance use services (Cieslik 2025).

For Fromm, the distortions of masculinity involved relate to purposeful confusion of potency (a capability to create and express oneself) with power (the tendency to dominate). Thus, Fromm saw a ‘lust for power’ as a corruption of masculinity, based on feelings of weakness rather than actual strength. Acceptance of the illusion that authoritarianism is a solution of disadvantage represents a transformation of the drive for life into a drive for destruction.

Writing long before a Trump presidency was ever imagined, Brian Easlea (1981) prophetically drew on the work of Marcuse and Fromm amongst others to reflect on the links between authoritarianism and a masculinity tied to anti‐feminism and misogyny. Easlea goes on to argue for a need for alternative politics framed by sensibilities congruent with ‘the desirability of increasing gentleness in all human relations’ (p219). de Sousa (1982) points out that Easlea's view challenges the authoritarian tendencies for oppressive masculine aggression in a clash between dystopian and utopian ideals. For Easlea, civilised living need not be impeded by the challenges and obstacles represented by masculinist aggressivity. In this regard, Margaret Atwood's classic feminist novel *The Handmaid's Tale*, far from being an imagined dystopian warning now seems to have been utilised as a blueprint for the demise of US democracy. Atwood herself wished for the book to serve as an anti‐prediction—if we can imagine the worst maybe we can stop it (Atwood 2017). Ultimately, the masculinist authoritarian conception of a new world order is a puerile prospectus (de Sousa 1982), resonant with contemporary critique observing Trump to often behave like a tantrum driven toddler.

Having described a range of critical thought on the problems of authoritarianism, we will now go on to address insights that may point to solutions, largely relational in character. First we consider some of our own research in the mental health field.

## 5 Reciprocal Insecurities in Relationships of Care

Some of our own work and ideas relating to the value of relational practices within mental health services, are congruent with thinking through the application of progressive psychosocial knowledge to the practice of political and social organising. Ironically for this piece, if we consider contemporary mental health services, concerned with the care of people who carry a personality disorder diagnosis, phenomenological research of ours has identified how insecurities in therapeutic encounters are shared between practitioners and patients. In a qualitative study of Crisis Resolution and Home Treatment services, data were gathered from 14 participants (seven staff, seven service users) using semi‐structured interviews and analysed using van Manen's phenomenological reflection. The findings illustrate ‘core structures’ of lived experience of participants. Anxieties associated with structural and inter‐personal vulnerabilities were illuminated as an essence of these crisis encounters for both service users and staff (Haslam et al. 2026). In this regard we suggest the reciprocal insecurities evident in the social relations of mental health services mirror the sort of insecurities that sustain authoritarianism in the political realm. Appreciation of both may be pivotal to gaining understanding of how to get out from under the heavy hand of authoritarianism and dominance and similarly the work towards democratising mental health care.

In some regards, these various fears about safety reflect many of the themes noted by the psychosocial scholars cited here. In a phenomenological study involving both nursing and service user participants, a major finding was how their relationships with each other, despite being about the transaction of care and support, were typified by reciprocal fears and anxieties. To some extent, this is unsurprising, when both parties to these service‐led encounters can feel vulnerable to imagined negative (or catastrophic) consequences or outcomes. Such fears are amplified within a society and services where concerns about risk are foregrounded, emphasised and not untypically exaggerated. Thus, it was not so surprising to hear both nurses and service users voice their anxieties about being or feeling unsafe. Individuals carrying a diagnosis of personality disorder seeking support from services at times of crisis expressed concerns about their personal acute vulnerability and anxieties surrounding the dual uncertainty over what the future holds for them or how they might be helped. In the extreme, these very real fears are about mortal safety—will people survive their crisis? Amidst a complexity of factors, these fears and uncertainties are markedly exacerbated by structural issues, such as economic austerity, that limit the range of available resources, including cuts to service provision. Staff shared in such concerns, including anxieties about staffing levels and continuities of service they could provide within constrained organisational resources.

Such uncertainties have been highlighted by Bauman (2000) as indicative of a wider social malaise of liquid modernity: staff and service users not confidently able to know from 1 day to the next who they will be supporting or receiving support from, who will be on the team (will it be established colleagues or agency staff?), what resources will be available and who will be operating services, who will be blamed if things go wrong, to name but a few of the many sources of uncertainty and thus insecurity. ‘Fear stalks the workforce, the liquid‐state fear of being replaced, moved or removed’ (Randall and McKeown 2013: 766).

Ultimately for sociologists like Bauman, this anxiety‐ridden state is toxic for relationships; being difficult to form or maintain good quality relationships in a context of such fast‐moving ambivalence and perpetual uncertainty. The uncertainties precipitated in public services are seen to be alienating for service users and staff, and consideration of such alienation connects scholars like Bauman with the aforementioned Frankfurt School critiques of capitalism. Similarly, Bauman points out that the sort of consumerist culture that characterizes late capitalism is constraining of human autonomy and solidarity, but awareness and resistance can potentially overcome these tendencies. For nurses, such awareness must include empathic appreciation of the manifest vulnerabilities and needs of service users and a corollary disavowal of unhelpful othering.

Nurses can maximise their capabilities to provide compassion and care for the ‘other’ by premeditatively facing up to liquid uncertainties through assertion of a more confident, agentic autonomy; the antithesis of an alienated professionalism (McKeown 2023a). Interestingly, it can be argued that mental health nursing's subordinate complicity with bio‐psychiatry may be explicable in similar terms to the Marcusian understanding of authoritarianism. In this scenario, nurses surrender to the dominance of bio‐psychiatry because to do so is reassuring in the face of a professional identity crisis: nurses who operate at the sharp end of psychiatric services, carrying out the dirty work of control and compulsion, can doubt their own value as relational beings and are aware of the relative lack of efficacy of their interventions for many people. Arguably, this breeds a deep‐seated professional insecurity and vulnerability that is to some extent assuaged by confident allegiance to the bio‐psychiatric episteme.

Our work utilises an appreciation of reciprocal insecurities to argue that care relationships must be built upon understanding, presence and space that allows the mental health nurse to interpret their experiences more broadly. It is a logical extension of this point to make a connection to more broadly cast critical thinking, so that the nurse is critically reflective and active across both the spaces of practice and wider social change. In this sense, nursing professionalism must also resonate with a broader set of political concerns, in ways that Bauman (2012: 167) would recognise as an embodiment of solidarity that is supportive of forms of advocacy and activism and expresses:

The inchoate and as yet inarticulate (but inborn and in the long run overwhelming) desire for a ‘good society’ which would make flesh the universal principles of justice.

How then should mental health nurses take on this challenge of resistance? Some possibilities are detailed below in terms of a preferred knowledge base for underpinning relational and democratised practices and concrete forms of action.

## 6 Democratising Care Practices and Politics

Some of the most persuasive commentary on mental health services, and how to change them for the better, is also intimately wrapped up with a more extensive political critique. The Marxist inspired criticism of 1960s and 1970s anti‐psychiatry furnished by the late great Peter Sedgwick (1982) arrives at a democratising prescription for change that targets both psychiatry and wider society as requiring the same relational transformations. Sedgwick's ideas have been revisited recently as particularly relevant to the anti‐democratic times we live in and for addressing more contemporary criticism of the perceived inadequacies of omnipresent, monoglot biological psychiatry (Beresford 2016; Spandler et al. 2016) and associated injustices (J. Fisher 2023). In a similar vein, Dyson (2024) also cites Sedgwick amidst a marshalling of the ideas of the revolutionary psychiatrist and anti‐colonial political activist Frantz Fanon. Like Sedgwick, Fanon, despite many contemporary admirers, including mental health nursing commentators concerned with social and practice change (Hopton 1995; McKeown and Wainwright 2019), has arguably been unjustly neglected with regard to acknowledgement and respect for his pioneering ideas for, amongst other things, democratised care services, de‐institutionalisation, culturally appropriate services, trauma informed care and a more progressive phenomenology of mental illness.

Dyson (2024) makes a convincing case for applying Fanon's ideas for transformative change in the present. Echoing the closing statement of Sedgwick's (1982) magnum opus, Psycho Politics, Dyson offers a cogent analysis of modern mental health services and how to change them. From a hopeful, Fanonian standpoint of substantial relevance to nurses concerned with the questions of what is to be done, and how to go about it, she declares:

Mental healthcare is not merely an opiate for the masses, but nor is it simply an unalloyed public good to be defended, restored and extended. Our recovery requires the radical transformation of both mental healthcare and the pathogenic social world …

Such radical change arguably requires the intervention of critically minded staff from within services, the activism of service users and survivors dissatisfied with services, and, potentially, democratically organised alliances between the two (McKeown et al. 2014). A reconsideration of nursing professionalism to embrace a more politicised professional identity could underpin the necessary activism of nurses both within services and across the wider political realm (McKeown 2023a). To some extent, such a political transformation of nursing professionalism has been held in check by the implicit conservatism of the historical quest for fully‐fledged professional status.

Yet, nurses have always been involved in resistance movements (McKeown 2019) and recent times and political turbulence have fomented a new cadre of politicised nurses concerned with a broad range of social justice issues and associated resistances, within and without healthcare workplaces, not least the pressing need to oppose the vicissitudes of right wing, authoritarian governments (e.g., Dillard‐Wright 2022, 2025; Essex et al. 2024; Goodman and Grant 2017; Hopkins‐Walsh et al. 2023; Lapum et al. 2025; Moth and McKeown 2016). Such developments are not the enemy of patient care or a distraction from it. Rather, the politically aware and active nurse is better placed to support service users in their existential struggles with either domineering bio‐psychiatry, such as navigating informed and consensual choices around medication and other treatments, or in advocating for social justice ends that can improve their material circumstances or prevent illness in the first place. We do not accept the premise that political neutrality is a more virtuous ethical or moral positioning for nurses. From this perspective, it is more ethical to be part of the resistance to dominance than to leave it in place by assuming a neutral standpoint.

To these ends, concrete examples of a politicised professionalism would include advocacy for more progressive and plural practice within care teams, particularly advocacy that challenges the dominance of psychiatry and uncritical use of coercive practices. It could also include more radical means such as conscientious objection to forced treatment or engagement in processes of truth and reconciliation (Gadsby and McKeown 2021; Spandler and McKeown 2017). We also urge nurses to become more active in collective organisations such as trade unions or social movements, either as activists or allies. The latter forms of advocacy and activism on a broader scale are congruent with nursing's professional commitments to social justice and awareness of the social and material determination of health. Arguably, these emergent forms of praxis share the sort of radical imagination that harks back to the spirit of earlier critical thinkers, such as those cited here, and advocating similar, relational democratic principles for how best to organise the necessary change.

That mental health nurses ought to occupy an active place within this politicised territory is for us unarguable. Not to take on the challenge could be profoundly self‐defeating in professional terms, not least as the profession faces other existential threats, such as a creeping genericisation of nursing that would dilute and devalue the importance of a distinct field of mental health nursing practice (e.g., Warrender et al. 2024). Interestingly, the organised resistance to genericisation also represents an opportunity to shift a defensive professional politics to a more radical questioning of the future epistemological basis, purpose and means by which such nursing is practised (McKeown 2023b; McKeown et al. 2026).

More democratic and relational care can offer an antidote to the insidious influence of demonising stereotypes, stigma and othering across society and also within therapeutic encounters. Pejorative conceptions of mental illness and personality disorder specifically interact subtly with the prominence of fear and danger driving governmental systems of control and paradoxical stoking of public fears (McKeown and Stowell‐Smith 2006). Despite decades of anti‐stigma campaigns, members of the public are reporting increased fear surrounding individuals identified as mentally ill and that such people are likely to place increasing demands upon services rather than recover (Mind 2025). Authoritarian responses are congruent with aiming to garner political capital via suppression of such fears. It is no accident that part of Trump's exercise of executive powers has been an institutionalising clampdown on people with mental health problems (Cieslik 2025).

## 7 Conclusion

We assert that forms of dominance and authoritarianism afflict the wider polity and mental health services alike, and a relational democratic standpoint is necessary for overcoming both. Our proposed solutions of advancing relational practices grounded in mutual recognition are resonant with the value placed on democracy held to in acts of resistance to authoritarianism. Indeed, antipathy to dominance as an oppressive force on the wider social scale is mirrored in the micro‐politics of resistance to an all‐pervasive bio‐psychiatry. We contend that progressive psychosocial ideas can be the basis of transformational change towards both better psychiatric services and a better world (see Dyson 2024; Sedgwick 1982). There is a long tradition of social psychiatry that has appreciated the psychosocial knowledge base drawn upon in this paper and developed forms of care and support steeped in democratic and relational principles. A prime example of this is the therapeutic communities tradition, which has been persuasively argued to constitute a social movement in its own right (Pskarris 2024). The inheritance of this tradition is a new relational practice movement that urges extensions of relational practice across all domains of public service (Haigh and Benefield 2024).

To achieve this, we must better understand what is going on. We have the intellectual tools, but mental health nurses and other progressives need to be part of raising consciousness and driving concerted action before it is too late. This demands a new professionalism that is not fearful of embracing a politicised identity. In this sense, a psychosocial understanding of the anxieties at play in the genesis and sustenance of authoritarianism can only be challenged if we are able to communicate this insight within collectives of resistance and overcome certain professional anxieties to embrace a more political framing for our nursing practice.

## 8 Relevance Statement

This paper is written by and for mental health nurses. The aim is to persuade mental health nurses that political insights are essential for nurses to navigate and challenge detriments to services and counter workplace alienation. A new politicised professionalism is congruent with nursing affinities for ethical care and relational practice.

## Conflicts of Interest

McKeown is an associate editor of the journal.

## Data Availability

Data sharing not applicable to this article as no datasets were generated or analysed during the current study.

## References

[jpm70132-bib-0001] Atwood, M. 2017. “Margaret Atwood on What ‘the Handmaid's Tale’ Means in the Age of Trump.” New York Times 10: 10.

[jpm70132-bib-0002] Bandy, L. 2017. The Dangerous Case of Donald Trump: 27 Psychiatrists and Mental Health Experts Assess a President. St. Martin's Press. 10.3399/bjgp18X699269 PMC6145972 30262624

[jpm70132-bib-0003] Beresford, P. 2016. “From Psycho‐Politics to Mad Studies: Learning From the Legacy of Peter Sedgwick.” Critical and Radical Social Work 4, no. 3: 343–355.

[jpm70132-bib-0004] Blake, C. , and C. Phelps . 1994. “History as Social Criticism: Conversations With Christopher Lasch.” Journal of American History 80, no. 4: 1310–1332. 10.2307/2080602.

[jpm70132-bib-0005] Burston, D. 2020. “Trump, Authoritarianism, and the End of American Democracy.” In Psychoanalysis, Politics and the Postmodern University, 87–107. Springer.

[jpm70132-bib-0006] Bussaja, J. 2024. “Is Mr. Trump A (White Narcissistic) Racist? SSRN.” 10.2139/ssrn.4847806.

[jpm70132-bib-0007] Cieslik, E. 2025. “Make Sanism Great Again: The Threat of Institutionalisation in the US.” Asylum Magazine 32, no. 4: 8–9.

[jpm70132-bib-0008] de Sousa, R. 1982. “Brian Easlea, “Science and Sexual Oppression”.” Philosophy in Review 2, no. 5: 214–217. https://journals.uvic.ca/index.php/pir/article/view/11144.

[jpm70132-bib-0009] Diamond, M. J. 2025. “A Psychoanalytic Perspective on Malignancies in the American Psyche: Containing Destructive Populism, Large Group Regression, and Cultism.” International Journal of Applied Psychoanalytic Studies 22, no. 3: p.e70008.

[jpm70132-bib-0010] Dillard‐Wright, J. 2022. “A Radical Imagination for Nursing: Generative Insurrection, Creative Resistance.” Nursing Philosophy 23, no. 1: p.e12371. 10.1111/nup.12371 34632696

[jpm70132-bib-0011] Dillard‐Wright, J. 2025. “The Perils of a Politics of Neutrality.” Witness: The Canadian Journal of Critical Nursing Discourse 7, no. 2: 1–3.

[jpm70132-bib-0012] Dyson, E. M. 2024. “From Critical Theory to Critical Therapy: Towards a Permanent Psycho‐Political Revolution Between Subjective and Objective Disalienation.” Philosophy & Social Criticism: 01914537241284541. 10.1177/01914537241284541.

[jpm70132-bib-0013] Easlea, B. 1981. Science and Sexual Oppression: Patriarchy's Confrontation With Woman and Nature. Weidenfeld and Nicolson.

[jpm70132-bib-0014] Essex, R. , L. Mainey , J. Dillard‐Wright , and S. Richardson . 2024. “Political Action in Nursing and Medical Codes of Ethics.” Nursing Inquiry 31, no. 4: e12658. 38973123 10.1111/nin.12658

[jpm70132-bib-0015] Fisher, J. 2023. “Epistemic Injustice: The Silenced Voices.” International Journal of Mental Health Nursing 32: 1186–1189. 10.1111/inm.13163. 37170848

[jpm70132-bib-0016] Fisher, M. 2009. Capitalist Realism: Is There no Alternative. Zero Books.

[jpm70132-bib-0017] Frances, A. 2017. “Misdiagnosing Donald Trump.” Journal of Mental Health 26, no. 5: 394. 28869403 10.1080/09638237.2017.1371845

[jpm70132-bib-0019] Fromm, E. 1937. “On the Feeling of Powerlessness.” Psychoanalysis and History 21, no. 3: 311–329.

[jpm70132-bib-0018] Fromm, E. 1957. “The Authoritarian Personality.” Mainstream 61, no. 48: 3–4.

[jpm70132-bib-0020] Gadsby, J. , and M. McKeown . 2021. “Mental Health Nursing and Conscientious Objection to Forced Pharmaceutical Intervention.” Nursing Philosophy 22: e12369. 10.1111/nup.12369. 34463024

[jpm70132-bib-0021] Goodman, B. , and A. Grant . 2017. “The Case of the Trump Regime: The Need for Resistance in International Nurse Education.” Nurse Education Today 52: 53–56. 28249198 10.1016/j.nedt.2017.02.013

[jpm70132-bib-0022] Haigh, R. , and N. Benefield . 2024. “The Development of a Relational Practice Movement.” In Relationships and Mental Health: Relational Experience in Distress and Recovery, edited by Z. Boden‐Stuart and M. Larkin , 259–282. Springer International Publishing.

[jpm70132-bib-0023] Haslam, M. , M. McKeown , E. Jones , G. Lamph , and K. Wright . 2026. “The Reciprocal Insecurity Paradox: A Phenomenological Analysis of the Crisis Encounter Between the Mental Health Nurse and Service User With Complex Emotional Needs.” Nursing Inquiry. 10.1111/nin.70125 PMC13268571 42296301

[jpm70132-bib-0024] Hopkins‐Walsh, J. , J. Dillard‐Wright , and B. B. Brown . 2023. “Nursing for the Chthulucene: Abolition, Affirmation, Antifascism.” Nursing Philosophy 24, no. 1: e12405. 36043247 10.1111/nup.12405

[jpm70132-bib-0025] Hopton, J. 1995. “The Application of the Ideas of Frantz Fanon to the Practice of Mental Health Nursing.” Journal of Advanced Nursing 21, no. 4: 723–728. 7797709 10.1046/j.1365-2648.1995.21040723.x

[jpm70132-bib-0026] Lapum, J. , C. Lee , R. Rahman , L. S. Nielsen , and H. Amanzai . 2025. “Impact of Trump's Executive Order on Nursing Research: The Shrouding of Racism Under the Guise of Equality.” Canadian Journal of Nursing Research 57, no. 1: 5–7. 10.1177/08445621251324053 40111919

[jpm70132-bib-0027] Laubender, C. 2026. “Why Sigmund Freud Is Making a Comeback in the Age of Authoritarianism and AI.” The Conversation. 10.64628/AB.9jnhgvdwe.

[jpm70132-bib-0029] Marcuse, H. 1955. Eros and Civilization. Beacon Press.

[jpm70132-bib-0030] Marcuse, H. 1964. One dimensional man. Beacon Press.

[jpm70132-bib-0028] Marcuse, H. , and D. Kellner . 1972. “The Historical Fate of Bourgeois Democracy.” In Towards a Critical Theory of Society, edited by D. Kellner , 163–186. Routledge.

[jpm70132-bib-0031] McKeown, M. 2019. “Love and Resistance: Re‐Inventing Radical Nurses in Everyday Struggles.” Journal of Clinical Nursing 29: 1023–1025. 10.1111/jocn.15084. 31661577

[jpm70132-bib-0032] McKeown, M. 2023a. “On the Bullshitisation of Mental Health Nursing: A Reluctant Work Rant.” Nursing Inquiry 31: e12595. 10.1111/nin.12595. 37622247

[jpm70132-bib-0033] McKeown, M. 2023b. “Editorial: Resisting Genericisation: Towards the Renewal of Mental Health Nursing.” Journal of Advanced Nursing 79: 3189–3191. 10.1111/jan.15598. 36797810

[jpm70132-bib-0034] McKeown, M. , M. Cresswell , and H. Spandler . 2014. “Deeply Engaged Relationships: Alliances Between Mental Health Workers and Psychiatric Survivors in the UK.” In Psychiatry Disrupted: Theorizing Resistance and Crafting the Revolution, edited by B. Burstow , B. A. LeFrancois , and S. L. Diamond . McGill/Queen's University Press.

[jpm70132-bib-0035] McKeown, M. , W. Sinclair , D. Collett , and H. Partington . 2026. “Mental Health Nursing and Politics: What Is To Be Done?” In Psychiatric and Mental Health Nursing: The Craft of Caring, edited by M. Chambers , Fourth ed. CRC Press.

[jpm70132-bib-0036] McKeown, M. , and M. Stowell‐Smith . 2006. “The Comforts of Evil: Dangerous Personalities in High Security Hospitals and the Horror Film.” In Forensic Psychiatry the Influences of Evil, edited by T. Mason . Humana Press.

[jpm70132-bib-0037] McKeown, M. , and J. Wainwright . 2019. “Echoes of Frantz Fanon in the Place and Space of an Alternative Black Mental Health Centre.” Critical & Radical Social Work 8: 323–338.

[jpm70132-bib-0038] Mind . 2025. “The big mental health report.” https://www.mind.org.uk/media/zianu2rn/mind_the‐big‐mental‐health‐report_digital4.pdf.

[jpm70132-bib-0039] Moth, R. , and M. McKeown . 2016. “Realising Sedgwick's Vision: Theorising Strategies of Resistance to Neoliberal Mental Health and Welfare Policy.” Critical & Radical Social Work 4, no. 3: 375–390.

[jpm70132-bib-0040] Pouncey, C. 2018. “President Trump's Mental Health—Is It Morally Permissible for Psychiatrists to Comment?” New England Journal of Medicine 378, no. 5: 405–407. 29281801 10.1056/NEJMp1714828

[jpm70132-bib-0041] Pskarris, K. 2024. “Therapeutic Communities as Social Movement.” University of Nottingham.

[jpm70132-bib-0042] Randall, D. , and M. McKeown . 2013. “Failure to Care: Nursing in a State of Liquid Modernity?” Journal of Clinical Nursing 23: 766–767. 10.1111/jocn.12441 24589229

[jpm70132-bib-0043] Sedgwick, P. 1982. Psycho Politics. Pluto Press.

[jpm70132-bib-0044] Semel, R. 2023. “Malignant Narcissists and Divisive Fabricators: Psychological Characteristics of Two US Presidents in a Tensely Divided 21st Century America.” EC Psychology and Psychiatry 12: 1–17.37424930

[jpm70132-bib-0045] Smith‐Frigerio, S. , and J. B. Houston . 2018. “Crazy, Insane, Nut Job, Wacko, Basket Case, and Psycho: Donald Trump's Tweets Surrounding Mental Health Issues and Attacks on Media Personalities.” In President Donald Trump and His Political Discourse, edited by M. Lockhart , 114–130. Routledge.

[jpm70132-bib-0046] Spandler, H. , and M. McKeown . 2017. “Exploring the Case for Truth and Reconciliation in Mental Health.” Mental Health Review Journal 22, no. 2: 83–94.

[jpm70132-bib-0047] Spandler, H. , R. Moth , M. McKeown , and J. Greener . 2016. “Editorial: Psychopolitics in the Twenty First Century.” Critical & Radical Social Work 4, no. 3: 307–312.

[jpm70132-bib-0048] Warrender, D. , C. Connell , E. Jones , et al. 2024. “Mental Health Deserves Better: Resisting the Dilution of Specialist Pre‐Registration Mental Health Nurse Education in the United Kingdom.” International Journal of Mental Health Nursing 33, no. 1: 202–212. 37788130 10.1111/inm.13236

[jpm70132-bib-0049] White, W. L. 2022. “A Prerequisite for Mad Liberation.” In The Routledge International Handbook of Mad Studies, edited by P. Beresford and J. Russo , 76–89. Routledge.

